# Microduplication and Microdeletion Syndromes Diagnosed Prenatally Using Single Nucleotide Polymorphism Array

**DOI:** 10.3390/jpm14030290

**Published:** 2024-03-08

**Authors:** Irina Ioana Iordănescu, Andreea Catana, Zina Barabas Cuzmici, Iuliana Chelu, Cristina Dragomir, Maria Militaru, Emilia Severin, Mariela Sanda Militaru

**Affiliations:** 1 Genetics Department, “Carol Davila” University of Medicine and Pharmacy, 020027 Bucharest, Romania; irina-ioana.iordanescu@drd.umfcd.ro; 2 Genetics Department Laboratory, Regina Maria, 011736 Bucharest, Romania; andreea.catana@reginamaria.ro (A.C.); zina_cuzmici_27@yahoo.com (Z.B.C.); iuliana.chelu@reginamaria.ro (I.C.); cristina.dragomir@reginamaria.ro (C.D.); sanda.militaru@umfcluj.ro (M.S.M.); 3 Genetics Department, “Iuliu Hațieganu” University of Medicine and Pharmacy, 400347 Cluj-Napoca, Romania; militaru.maria@elearn.umfcluj.ro

**Keywords:** microdeletion/microduplication syndromes, SNP array, prenatal genetic diagnosis, abnormal ultrasound

## Abstract

We present a series of microdeletion and microduplication syndromes (MMSs) observed in our clinical practice over a three-year period from 2020 to 2023. Microdeletion and microduplication syndromes, characterized by chromosomal deletions or duplications of less than five megabases, pose challenges in terms of diagnosis, especially prenatal and clinical management. Clinically, MMSs encompass a broad spectrum of manifestations, ranging from intellectual disability and developmental delays to congenital anomalies, facial dysmorphisms, and neurobehavioral abnormalities. Notable examples include well-characterized syndromes such as DiGeorge syndrome (22q11.2 deletion), Prader–Willi syndrome (15q11–q13 deletion), and Williams syndrome (7q11 deletion). Our study focuses on the genetic foundations and prenatal ultrasound findings of these syndromes, with an emphasis on cases associated with intellectual disability. Using SNP array technology, we delve into the evolving landscape of diagnostic methods, providing a nuanced understanding of copy number variations (CNVs) and their implications. Prenatal diagnosis allows for the early detection of MMSs, enabling parents and healthcare providers to make informed decisions about the pregnancy and plan for appropriate medical care and interventions. Beyond theoretical considerations, our article bridges the gap between research and practical application by offering insights derived from clinical cases. Through the presentation of specific cases, we aim to contribute valuable data to the broader discourse on MMSs, fostering knowledge exchange and enhancing the medical community’s awareness of these complex genetic conditions.

## 1. Introduction

Microdeletion and microduplication syndromes (MMSs) represent a diverse group of genetic disorders characterized by chromosomal deletions or duplications of less than five megabases (Mb). As highlighted in a comprehensive paper published in 2022 [1], which listed 192 such syndromes, examples abound, including the well-known 15q11–q13 deletion associated with Prader–Willi and Angelman syndromes, the 17p11 deletion linked to Smith–Magenis syndrome, the 7q11 deletion related to Williams–Beuren syndrome, and the 22q11.2 deletion associated with DiGeorge syndrome [2]. The spectrum of MMSs encompasses a broad range of clinical manifestations, from multiple congenital anomalies and developmental delays in microdeletion syndromes to relatively milder phenotypes often observed in reciprocal microduplication syndromes. Despite their clinical heterogeneity, both microdeletion and microduplication syndromes pose diagnostic challenges, especially given their predominantly spontaneous occurrence, accounting for approximately 5% of patients with unexplained intellectual disability [3]. It is important to note that while the literature suggests that for every known microdeletion syndrome, there exists a reciprocal microduplication syndrome, where a gene segment is both deleted and duplicated [4], the disparity in reported cases hints at complexities yet to be fully elucidated, possibly influenced by variations in clinical presentation and severity. Advancements in diagnostic methodologies, such as SNP array technology, chromosomal microarray analysis (CMA), and next-generation sequencing (NGS), have facilitated a deeper understanding of copy number variations (CNVs) associated with MMSs, aiding in the characterization of both benign and pathogenic variants and shedding light on the underlying genetic mechanisms driving recurrent syndromic occurrences [5].

Microdeletions and microduplications constitute a distinctive category of genetic disorders that can profoundly affect an individual’s health and development. Although each microdeletion and microduplication syndrome is rare individually, their collective occurrence is surprisingly common. Additionally, it is noteworthy that MMSs might be overlooked in certain instances, where they present with an apparently normal phenotype, making it challenging to locate precise prevalence data in the medical literature.

This study endeavors to bridge the gap between theoretical research and practical application by offering insights derived from clinical cases encountered in our practice. Through the analysis of specific cases, we aim to contribute substantive data to the ongoing discourse surrounding MMSs, fostering knowledge dissemination and augmenting the medical community’s comprehension of these intricate genetic conditions.

## 2. Materials and Methods

In our clinic, prenatal diagnosis utilizing the SNP array technique was employed to identify microdeletion and microduplication syndromes, loss of heterozygosity, and the detection of uniparental disomies (UPDs) and triploidies. This approach reflects the common practice observed in both public and private laboratories, where individual choices regarding the technique and platform used for analysis are often restricted to a single type due to financial constraints. While SNP array technology may initially have higher upfront costs compared with some other diagnostic techniques, its high-throughput nature and ability to provide comprehensive genomic analysis can ultimately result in cost savings by reducing the need for multiple tests and increasing diagnostic yield.

Over the span of three years, from 2020 to 2023, a thorough examination was carried out on 673 prenatal samples. All samples were obtained from amniotic fluid, with one exception (a 12-week pregnancy that had a chorionic villus sampling), originating from pregnancies occurring between 16 and 20 weeks. The investigation resulted in the identification of 93 abnormal cases, with 21 of them being specifically linked to microdeletion or microduplication syndromes. The primary indication for testing was abnormal ultrasound findings, an NIPT test with high risk, a double test with high risk, or advanced maternal age.

For quality control and to ensure accurate results, Quantitative Fluorescent Polymerase Chain Reaction (QF-PCR) was conducted using the Devyser v3 compact kit. This kit is specifically designed for the amplification, detection, and analysis of chromosome-specific DNA sequences known as genetic markers or Short Tandem Repeats (STRs). A total of twenty-six genetic markers, labeled with fluorescent probes, were analyzed, and quantified using an automated genetic analyzer (ABI 3500, Applied Biosystems, Waltham, MA, USA). The interpretation of results was performed using GeneMapper software (4.4.1, Applied Biosystems, Waltham, MA, USA). This test aimed to exclude maternal contamination and provided a rapid screening method to identify common aneuploidies. None of the samples presented maternal contamination.

In addition, the hybridization method utilized the Affymetrix CytoScan 750 K platform with a resolution of 100 kb (Applied Biosystems, Waltham, MA, USA). This platform employs a hybrid design enabling the detection of copy number variations (CNVs), precise determination of breakpoints, and identification of loss of heterozygosity (LOH). With 750,000 markers for copy number and 200,000 genotype-able Single Nucleotide Polymorphisms (SNPs), it provides high-resolution copy number analysis and accurate breakpoint estimation. The databases employed include Chromosome Analysis Suite (ChAS) Software (V3.1), NCBI Build GRCh38 (hg38), ClinVar, OMIM (Online Mendelian Inheritance in Man), NCBI, and DECIPHER.

It is important to note that while the SNP array technology used in this study offers high-resolution analysis, it does not detect balanced translocations, inversions, point mutations, or low-grade mosaicism. The limit for SNP array detection of mosaicism is 15–20%.

## 3. Results

### 3.1. Case Reports

**Figure 1:**
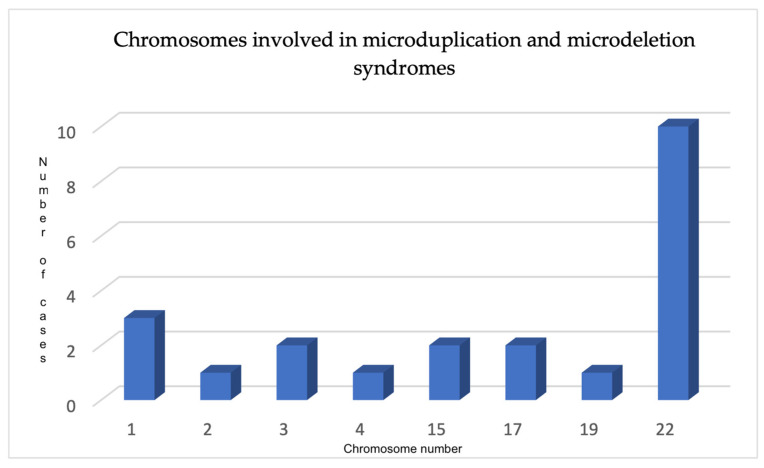
Chromosomes involved in MMSs in our clinic.

The microarray analysis involved the examination of all chromosomes; however, only specific chromosomes were found to be involved in microdeletion and microduplication syndromes. These included chromosome 1 (involving two pregnancies of the same patient and the patient itself), chromosome 2 (in one case), chromosome 3 (in two cases), chromosome 4 (in one case), chromosome 15 (in two cases), chromosome 17 (in two cases), chromosome 19 (in one case), and chromosome 22 (in ten cases), as depicted in Figure 1.

The 22q11.2 microdeletion syndrome stands out as the most prevalent microdeletion, occurring in approximately 1 in 4000–6000 live births [6]. The phenotype may include DiGeorge syndrome, velocardiofacial syndrome, or conotruncal anomaly face syndrome. While most patients exhibit a 3 Mb deletion, smaller deletions have also been documented. Variability in phenotype, observed within and among families, is attributed to haploinsufficiency of genes located at 22q11.2. Primary symptoms include thymus dysfunction, congenital heart malformations (such as tetralogy of Fallot), immunodeficiency, and intellectual disability [7].

In our clinic, deletions ranged between 735 kb and 2.9 Mb. The frequency of 22q.11.2 microduplication syndrome is estimated at 1/700 of the population with intellectual disability, but its real frequency may be greater due to its variable phenotype, which includes congenital heart disease, gastrointestinal problems, endocrinological dysfunction, or neurological complications [8].

Genetic counseling was offered to most patients before amniocentesis. Despite written recommendations for parental testing in all cases, only three couples opted for the test. Parental testing, conducted using MLPA (multiplex ligation-dependent probe amplification) or SNP array, revealed that in two cases, the deletion was inherited from the mother, and in the third case, the duplication was inherited from the healthy father. One of the two mothers had suspicions of 22q11.2 deletion syndrome after genetic counseling. Following the results, all patients who underwent genetic testing received counseling and chose to continue with the pregnancy.

Unfortunately, there is no further information about patients who did not follow up with the consultation.

This underscores the significance of genetic counseling both before and after testing and highlights the clinical geneticist’s access to pertinent clinical information.

### 3.2. Case 1

**Figure 2:**
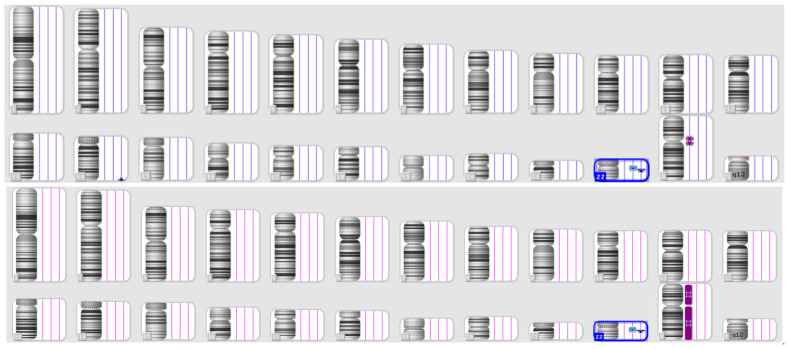
The SNP array results of the fetus (**above**) and the father (**below**)—Case 1.

In the first case, a patient at 16 weeks of pregnancy underwent an early NIPT test, revealing a high risk of a microduplication on chromosome 22. Subsequently, amniocentesis and SNP array testing were recommended. The test results confirmed the NIPT findings, indicating a female fetus with a 4 Mb duplication on chromosome 22q11.23q12.1, spanning positions 2,321,3491–2,727,1834 (Figure 2).

Individuals carrying this microduplication may exhibit a wide range of symptoms, including intellectual disability, learning difficulties, developmental delay, small stature, hypotonia, or even an apparently normal phenotype [9].

Following genetic counseling, the couple opted to assess whether the microduplication was inherited or de novo. Results indicated that the microduplication was inherited from a phenotypically normal father, and the couple chose to proceed with the pregnancy after genetic counseling.

### 3.3. Case 2

**Figure 3:**
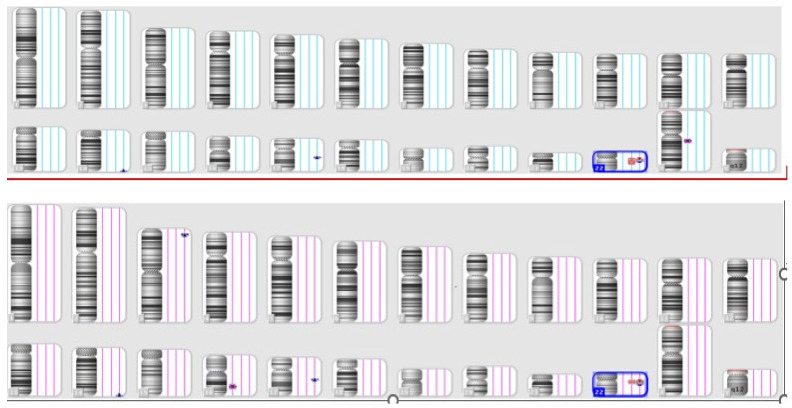
The SNP array result of the mother (**above**) and the fetus (**below**)—Case 2.

This patient presented to the clinic following abnormal findings in the ultrasound screening, which included bilateral pyelectasis, choroid plexus cysts, and a heart malformation. The result of the SNP array revealed a 2,9 Mb deletion on chromosome 22 between 18,166,088 and 21,110,475, inherited from the mother. Based on SNP array results, DiGeorge syndrome was diagnosed (Figure 3). During genetic counseling, the physician observed the mother’s hypernasal speech, prompting a recommendation for the mother to undergo testing simultaneously with the fetus. DiGeorge syndrome exhibits significant phenotypic variability, even among family members, encompassing congenital heart defects, palatal abnormalities, immune deficiency, facial dysmorphism, and intellectual disability [10]. Despite the fetus having a more severe cardiac malformation than the mother, the couple opted to continue with the pregnancy.

### 3.4. Case 3

**Figure 4:**
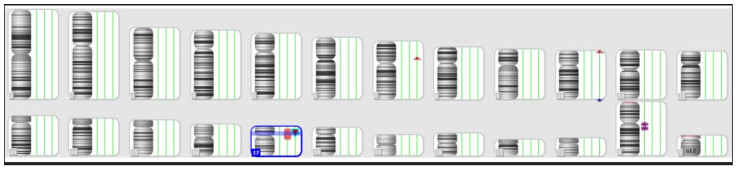
The SNP array result of the fetus—Case 3.

The third case involves a patient with an ongoing 22-week pregnancy, where the ultrasound revealed tetralogy of Fallot. Amniocentesis and SNP array were conducted, revealing a female molecular karyotype with a 3.5 Mb deletion on the short arm of chromosome 17p11.2, spanning between 16,860,383 and 20,420,309 (Figure 4). This deletion, classified as pathogenic in databases, encompassed 41 OMIM genes, including RAI1 and FLCN. The microdeletion is associated with Smith–Magenis syndrome, an autosomal dominant disorder characterized by the absence or pathogenic variant of the RAI1 gene. In this case, the deletion also included the FLCN gene, potentially leading to a more severe phenotype with overlapping features of Birt–Hogg–Dubé syndrome.

Smith–Magenis syndrome is characterized by facial dysmorphism, global developmental delay, congenital heart defects, sleep and behavior disturbances, and childhood-onset abdominal obesity [11]. Birt–Hogg-]–Dubé syndrome exhibits significant variability among family members and is characterized by cutaneous manifestations (fibrofolliculomas, acrochordons), pulmonary cysts, and renal tumors [12]. The patient did not seek consultation at our clinic either before or after the genetic test was performed.

### 3.5. Case 4

**Figure 5:**
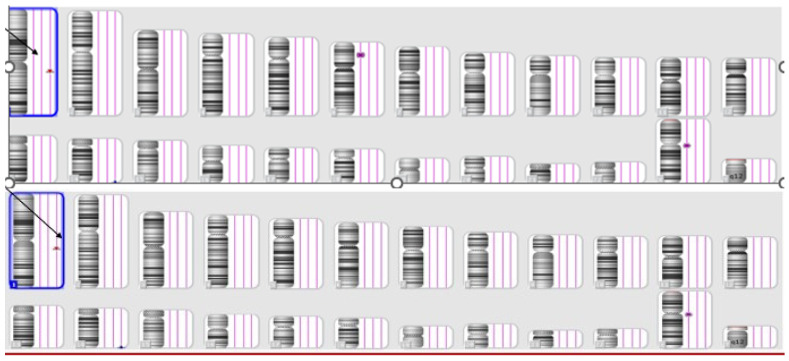
The SNP array result of the mother (**above**) and the fetus (**below**)—Case 4.

Presented here is a case of a 22-week pregnancy with bilateral radius agenesis detected during ultrasound. Urgent amniocentesis and SNP array were performed, revealing a female molecular karyotype with a 453 kb microdeletion on the long arm of chromosome 1q21.1 between 145,605,588 and 146,058,926 (Figure 5). Considering the clinical data and SNP array results, the diagnosis of TAR (Thrombocytopenia-absent radius syndrome) was presumed. The couple, given the ultrasound findings and advanced pregnancy, opted to terminate the pregnancy without further sequencing of the RBM8A gene.

TAR syndrome is characterized by a variable degree of radial defects and neonatal thrombocytopenia, resulting from a heterozygous deletion of one allele on chromosome 1 and another mutation in the RBM8A gene [13].

After a year, the same couple underwent another pregnancy and opted for an NIPT test, revealing a high risk of monosomy X. Considering the patient history, CVS and SNP array were performed, uncovering a female fetus with a 576 kb microduplication on the long arm of chromosome 1q21.1 between 145,605,588 and 146,057,946 The couple sought genetic counseling and decided to undergo testing to determine if one of them was a carrier of the microdeletion. After testing, it was discovered that the mother carries the microdeletion. Subsequently, during both pregnancies, she transmitted it to the fetuses. Given that the mother exhibited a phenotypically normal appearance, the couple chose to continue the pregnancy and undergo ultrasound screening for absent radius.

### 3.6. Case 5

**Figure 6:**
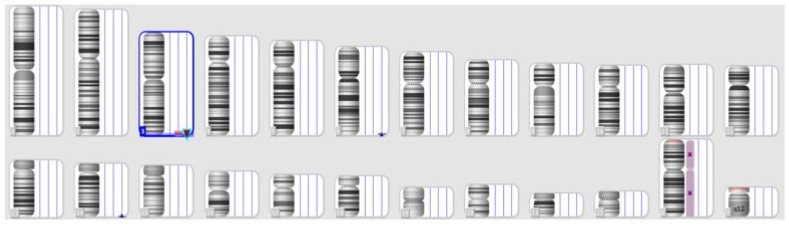
The SNP array with a 1.6 Mb microdeletion on the long arm of chromosome 3.

The last case involves a patient for whom the only clinical information provided to the laboratory was advanced maternal age. Subsequently, amniocentesis and SNP array were conducted, revealing a male karyotype with a 1.6 Mb microdeletion on the long arm of chromosome 3q29, encompassing 19 OMIM genes (Figure 6).

The 3q29 microdeletion exhibits unknown penetrance and a broad range of symptoms, including mild to moderate intellectual disability, autism, ADHD, schizophrenia with earlier onset than the general population, feeding difficulties, ocular manifestations, or congenital heart defects. However, there are also individuals with an apparently normal phenotype [14].

During genetic counseling, it was discovered that the couple had a previous child with autism but without a genetic diagnosis. Faced with the unknown risk of the second child developing autism, the couple chose not to proceed with the pregnancy. However, they were advised to undergo additional testing to understand the cause of autism in the first child. The initial recommendation was to test all three family members using a SNP array. If the SNP array yields negative results, further testing with whole exome or whole genome sequencing would be conducted to determine if one of the parents carries the microdeletion.

## 4. Discussion

The cases described above represent individuals who underwent genetic counseling either before or after testing in our practice. However, it is not uncommon for genetic laboratories to receive only the minimal required data for testing, and we often lack information on whether other patients received genetic counseling. For instance, in one case, we identified an incidental finding during an amniocentesis performed at 22 weeks of pregnancy for intrauterine growth restriction (IUGR). The fetus was found to have a 1.3 Mb microduplication on chromosome 17, including the PMP22 gene. To our knowledge, this patient did not undergo genetic counseling at our clinic.

Another notable occurrence in our study involves the detection of 15q11.2 microduplication, which is commonly associated with Prader–Willi and Angelman syndromes according to the cited literature. However, in our practice, we encountered two cases of chromosome 15q microduplication. One case involved a 2.5 Mb microduplication at 15q13.2q13.3, while the other involved a 1.2 Mb microduplication at 15q11.2. These duplications exhibit a variable phenotype as described in OMIM (608,636), including features such as autism, developmental delay, ataxia, and behavior problems.

The size and the location of microdeletion and microduplication syndromes vary, but they usually involve contiguous genes (the term describes conditions associated with microdeletions but can also include instances of microduplications where genes are clustered together [4]) with a critical region involved. They are not related to maternal age; clinically relevant microdeletion and microduplication syndromes occur in 1.7% of all structurally normal pregnancies, and most of these copy number variations (CNVs) are recurrent and well-associated with several syndromes. Non-invasive prenatal screening (NIPT) is also addressed for these types of structural rearrangements as well as for trisomies, but the test poses some challenges due to their small dimension and the proportion of cfDNA. The detection rates and false-positive and positive predictive values vary widely between the test and the technology they use [15].

Although NIPT tests are used widely in our country as a screening test starting from 10 weeks of pregnancy before the first-trimester ultrasound can be performed, for their high accuracy, specificity, and sensitivity for trisomy 21, 18 and 13, their accuracy may vary for aneuploidies of sex chromosomes or structural rearrangements >10 Mb. So, confirmatory testing may be necessary in some cases. NIPT is not usually used as a method of screening for microdeletion or microduplication syndromes, but it can reveal a high-risk result for them as the test requested also evaluates MMSs. A high-risk result must be confirmed. In one study, from six high-risk results, only one was confirmed with a diagnostic of microdeletion [16].

There have been numerous other studies about the prevalence of MMSs, but most of these studies have been performed in postnatal pediatric settings, where the phenotype can be very well observed. The same does not apply in the prenatal world, where ultrasound is the only method to observe the phenotype partially since there can be no evaluation of the intellectual disability.

In 2018, another study tried to determine the prevalence of MMSs with genomic microarray due to the non-vast literature approaching the subject, exempt for 22q11.2 microdeletion syndrome. That study determined the prevalence of 1q21.1 microdeletion syndrome at 0.015% (1/6882) with the prevalence of 1q21.1 microduplication syndrome at 0.016% (1/6309), the prevalence of 16p11.2 microdeletion syndrome at 0.03% (1/3021), and the prevalence of 16p12.2 microduplication syndrome at 0.023% (1/4216) [17]. Although the prevalence of one microdeletion or microduplication syndrome is rare, the total prevalence of MMSs is higher, and the prenatal diagnosis for them could further validate this research.

In 2019, a study was published about DiGeorge syndrome stating that in 5464 pregnancies, 16 were with 22q11.2 microdeletion and 11 with 22.q11.2 microduplication. The cases with deletion presented more ultrasound structure defects as congenital heart defects, multiple congenital anomalies, or kidney problems, in contrast to the cases with duplication that presented fewer ultrasound modifications [18]. We consider our study to be in accordance with the data presented in the previous study.

A Brazilian study revealed that in 5778 individuals with general neurodevelopmental disorder and/or congenital abnormalities without evident cause, CNVs were found in 1886 individuals classified as pathogenic, likely pathogenic, and VUS (41%) [19].

The application of SNP array widely as a first trier diagnostic test and a gold standard for the detection of CNV has improved the detection rate of microduplications and microdeletion syndromes as well as other structural rearrangements for cases with ultrasound findings or high-risk noninvasive testing. A trio approach could help the families decide faster as time is of the essence in a prenatal setting, and from our experience, the families where the microdeletion and microduplication were inherited decided to continue with the pregnancy. Also, it informs the patients about recurrence risk in a future pregnancy. With the growing use of whole-exome sequencing in prenatal care, or even trio testing, this could become, in future years, the new first-trier test, but there are still countries where access to this technology is not available to everyone, and the recommendation for this testing is not common in clinical practice.

While NGS technology is the best method to detect single nucleotide variants (SNVs) and small deletions and insertions, it is still challenging to detect CNVs due to short read lengths or CG-rich content areas [20].

The clinical interpretation can still prove challenging, especially in a prenatal setting where the possibility of a variant of unknown significance is found. Generally, the ACMG (American College of Medical Genetics) criteria and guidelines are used. These guidelines, regarding categorization and reporting, currently exist only for postnatal microarrays. In the prenatal setting, reporting is not standardized between laboratories and countries, the differences being about CNVs with morbid OMIM genes, the size of the CNV, penetrance, carrier status, or ROH (regions of homozygosity) [21].

Our study advocates for standardized reporting practices, particularly in prenatal settings, to address the interpretative complexities and ensure consistency across laboratories and countries.

Given the substantial intrafamilial variability and expressivity of syndromes, genetic counseling becomes challenging, particularly for novel or poorly described microdeletion or microduplication syndromes. Multidisciplinary care for children with these syndromes is essential, considering the wide spectrum of congenital malformations, necessitating long-term follow-up, especially for diagnoses with novel or poorly described syndromes.

This study described our findings of microdeletion and microduplication syndromes from a large cohort of patients that underwent amniocentesis for prenatal diagnosis, although in many cases, the ultrasound or the NIPT test indicated other syndromes such as trisomy 21 or diaphragmatic hernia. In other instances, the ultrasound revealed a congenital heart malformation.

Prenatal diagnosis allows for the early detection of MMSs, enabling parents and healthcare providers to make informed decisions about the pregnancy and plan for appropriate medical care and interventions. Despite advances in prenatal diagnostic techniques, there are still challenges and limitations associated with detecting MMSs prenatally. These may include the need for invasive procedures such as amniocentesis or chorionic villus sampling (CVS), as well as the possibility of uncertain or inconclusive results. Prenatal diagnosis of MMSs raises ethical considerations related to reproductive autonomy, informed decision-making, and the potential psychological impact on parents.

The exact prevalence of microdeletion and microduplication syndromes can vary depending on several factors, including the studied population, the diagnostic methods used, and the inclusion criteria in studies. Because these syndromes can be individually rare and may be underdiagnosed in some cases, it may be more difficult to find precise prevalence data in the medical literature. Our study conducted at a tertiary clinic in Bucharest, Romania, provides information about MMSs in a population from the Southeastern European region. While this study aligns with some findings from previous studies, there might be variations in methodologies, sample sizes, and populations that could influence direct comparisons. In our practice, microdeletion and microduplication syndromes had a 3.12% prevalence, a slightly increased percentage than in the literature, taking into consideration the frequency of all MMSs together and not the frequency of each MMS separately. While SNP array technology may initially have higher upfront costs compared with some other diagnostic techniques, its high-throughput nature and ability to provide comprehensive genomic analysis can ultimately result in cost savings by reducing the need for multiple tests and increasing diagnostic yield.

Future research endeavors should continue to address the challenges in this field, especially in the prenatal setting where phenotypic features are not always available on ultrasound, ultimately enhancing the quality of care provided to individuals and families affected by these syndromes.

## 5. Conclusions

In our clinic, the SNP array served as a valuable initial diagnostic tool for microdeletion and microduplication syndromes, providing clinicians and patients with crucial insights into chromosomal abnormalities that may impact fetal development. By detecting copy number variations (CNVs) with high accuracy and resolution, the SNP array aided in the early identification of genetic anomalies, allowing for timely interventions and informed decision-making by expectant parents.

Genetic counseling played a crucial role both before and after testing, serving as a cornerstone of prenatal care for families facing the possibility of genetic disorders. Pre-test counseling helped parents understand the implications of genetic testing, including the potential outcomes and limitations of the SNP array. Post-test counseling provided support and guidance in interpreting test results, addressing any uncertainties or concerns, and facilitating informed decision-making regarding the current pregnancy and about the recurrence risk of future pregnancies The possibility of testing other relatives at risk was discussed as well.

As diagnostic techniques continue to advance, with the adoption of technologies like SNP array and WES in prenatal settings, there is a growing need for more standardized prenatal guidelines. Standardization ensures consistency in testing methodologies, result interpretation, and reporting practices across different laboratories and countries, enhancing the reliability and comparability of prenatal genetic testing results, especially regarding the frequent variants of unknown significance found that pose difficult challenges for the laboratory team to interpret, report, and eventually reclassify. By establishing clear and uniform guidelines and follow-up testing, healthcare providers can deliver more effective and equitable prenatal care, ensuring that all expectant parents receive comprehensive information and support to navigate the complexities of genetic testing and make well-informed decisions for the health and well-being of their families.

## Data Availability

The data are unavailable due to privacy or ethical restrictions.
